# Imatinib-Induced Bone Marrow Aplasia in Chronic Myelogenous Leukemia

**DOI:** 10.7759/cureus.61176

**Published:** 2024-05-27

**Authors:** Ratika Dogra, Vallabh Dogra, Abhay R Shelke

**Affiliations:** 1 Internal Medicine, Bon Secours Mercy Health, Toledo, USA; 2 Hematology Oncology, Bon Secours Mercy Health, Toledo, USA

**Keywords:** chronic myelogenous leukemia, hypocellular bone marrow, myelosuppression, pancytopenia, imatinib, tyrosine kinase inhibitors

## Abstract

Imatinib is a tyrosine kinase inhibitor (TKI) and is a commonly used medication for treatment of chronic myelogenous leukemia (CML). Aplastic anemia is a very uncommon complication of Gleevec, and only a few cases are reported in the literature. We present a case of a 63-year-old Asian female who was initiated on imatinib for treatment of CML with good response in cell counts. Four months after Gleevec initiation, the patient was admitted to the hospital with extreme fatigue and noted to have severe pancytopenia. Patient received multiple blood transfusions. Finally, the patient underwent bone marrow biopsy, which showed concern for aplastic anemia with marked hypocellular bone marrow. Gleevec was held, blood counts were monitored, and supportive care was given. Patient had slow recovery of her blood counts. There remains scarcity of data on this topic and no criteria exist to predict the myelosuppression with TKI therapy. Our case report aims to reemphasize the need for increased research on myelosuppression with TKI therapy.

## Introduction

Imatinib is the first-generation and most widely used tyrosine kinase inhibitor (TKI) in chronic myelogenous leukemia (CML) treatment. It is generally well tolerated but can cause myelosuppression in various degrees. Early myelosuppression can be secondary to decreased reserve or lack of recovery of normal stem cells. Prolonged suppression has rarely been reported [1]. Here we report a rare case where imatinib was initiated for treatment of CML in the first line setting and was associated with significant hematological recovery with rapid improvement in blood counts within two months. However, the patient developed significant pancytopenia within four months of therapy and imatinib was held.

## Case presentation

A 63-year-old Asian female with a known history of peptic ulcer disease and diastolic heart failure was admitted to the hospital with complaints of fatigue. She was noted to have significant elevation of white blood cell count (WBC) of 380x10^9^/L, hemoglobin (HB) of 3.9 gm/dl, and platelet count (PC) 436x10^9^/L and imaging revealed a concern of splenomegaly. Bone marrow biopsy showed leukocytosis with absolute neutrophilia and left shifted maturation, monocytosis, eosinophilia and basophilia. Rare circulating blasts were identified. Chromosome study showed abnormal karyotype with T(9,22) and FISH was positive for BCR-ABL fusion. BCR-ABL quantitative analysis was high positive. Finally, diagnosis of CML-chronic phase was established. Due to elevated WBC, patient was started on hydroxyurea and within one week patient was initiatedLDH on imatinib treatment at 400 mg. Her blood counts responded reasonably well. The patient was seen two months after starting therapy, and at that time, her WBC was 3.5x10^9^/L, HB was 9.9 gm/dl, and platelets were 128. x10^9^/L. The decision was to continue with imatinib at the same dosage level.

**Figure 1:**
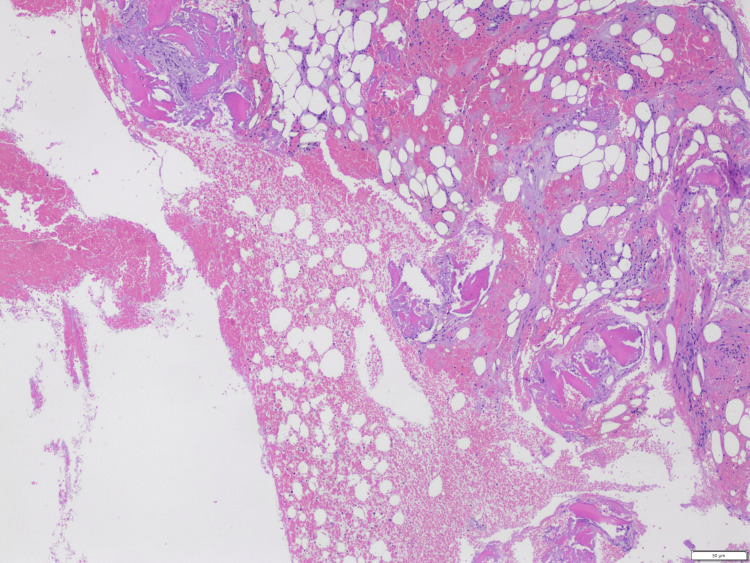
Bone marrow biopsy with hematoxylin and eosin staining showing hypocellular bone marrow

The patient subsequently presented to the hospital four months from her initial diagnosis with poor appetite, fatigue, and generalized body aches. Blood work showed severe pancytopenia, HB decreased to 3.1 gm/dl, WBC was 0.4x10^9^/L and platelets were down to 21x10^9^/L. She also tested positive for COVID-19 but did not require any oxygen support. The patient received multiple blood transfusions, and imatinib was withheld. The patient had reticulocyte% of 1.6, haptoglobin 321, lactate dehydrogenase (LDH) 168, ruling out hemolytic anemia, and fecal stool occult blood was negative, ruling out gastrointestinal bleeding. Patient’s blood counts remained low and patient received supportive care with blood transfusions. Our evaluation suggested possible severe bone marrow suppression secondary to drug or infection. Another possibility was the transformation of the CML to myelodysplastic syndrome or acute leukemia. She underwent second bone marrow biopsy showing markedly hypocellular marrow with scattered lymphocytes and plasma cells (Figure 1). There was no evidence of increased blasts, dysplastic changes, myelofibrosis and overall findings were suggestive of aplastic anemia likely secondary to treatment. There was not enough sample for chromosomal analysis. The patient continued to follow up in the hematology office and continued to have an improvement in blood counts after Imatinib was stopped (Table 1). BCR-ABL quantitative analysis remained high positive.

**Table 1:** Trend of WBC, HB and PC in our patient Month 0 is initiation of Gleevec therapy after the initial diagnosis of CML; Months 1, 2 show improvement of cell counts after therapy; Month 4 is when patient presented to hospital and Gleevec was held; Months 5, 6 represent improving cell counts after withholding Gleevec therapy. WBC: White blood cell count; HB: Hemoglobin; PC: Platelet count

	Month 0 at diagnosis	Month 1	Month 2	Month 4	Month 5	Month 6
WBC (4-11x10^9^/L)	380.7	6.6	3.5	0.4	0.7	2.2
HB (11.7-15.5 g/dl)	3.9	8.2	9.9	3.1	6.5	10.2
PC (150-400 x10^9^/L)	436	445	128	21	22	146

## Discussion

Imatinib, with brand name Gleevec, is a TKI that received US Food and Drug Administration (FDA) approval in 2001. Imatinib is a 2-phenylamino-pyrimidine derivative protein. TKIs are breakthrough medications which changed the therapeutic landscape of CML treatment. They are used to manage and treat malignancies like CML and gastrointestinal stromal tumors (GISTs) [2]. They were initially targeted to the platelet-derived growth factor receptor. Subsequently noted to inhibit other protein kinases like c-kit and BCR-ABL fusion protein [3].

CML is a myeloproliferative disorder with abnormal pluripotent stem cells with 90-95% cases having characteristic t (9; 22) translocation. This translocation fuses sequences of the BCR gene on chromosome 22 with regions of the ABL 1 gene from chromosome 9 resulting in the formation of BCR-ABL chimeric protein. This protein has enhanced tyrosine activity [4]. TKIs selectively inhibit several tyrosine kinases central to pathogenesis of human cancer.

Common side effects of imatinib include fluid retention, peripheral edema, nausea, vomiting, musculoskeletal pain, fatigue, rash, fever, hypokalemia, neutropenia, transaminases, anorexia, and photosensitivity. Serious side effects include ascites, pleural and pericardial effusion, myelosuppression, exfoliative dermatitis, acute renal failure, and tumor lysis syndrome [5]. Myelosuppression is significantly more common with Imatinib therapy in CML patients compared to patients of GISTs [6].

Aplastic anemia is an uncommon complication. Very few cases of imatinib induced aplasia are reported in literature. Transient cytopenia can occur commonly during Imatinib therapy in CML however higher grade of myelosuppression (Grades 3-4) can occur in initial stages of treatment and the risk declines with longer therapy duration [7]. The initial aplasia can occur due to eradication of CML clones, which initially compose the cells in the bone marrow. TKIs affect the proto-oncogene c-kit, which affect normal hematopoiesis, resulting in unwanted suppression of progenitor stem cells [8]. With continuation of effective therapy, normal blood count returns as normal hematopoiesis is restored in the marrow and CML clones are reduced [9]. In patients with aplasia biopsy of bone marrow show significant hypocellularity and fatty tissue without evidence of myelofibrosis [10].

A study by Lokeshwar et al. described a case where the patient received imatinib and developed aplasia, this patient received busulphan and IFN-a in the past [11]. Sumi et al. also reported similar findings where the patient was treated with interferon-alpha, hydroxyurea, and busulfan before imatinib mesylate treatment [12]. The mechanism underlying this myelosuppression is not fully understood. Old age, advanced disease and history of previous treatment with busulfan or intereferon alfa are noted to have increasing risk of myelosuppression [13]. Asian patients are sometimes intolerant to the recommended dose, and TKIs can achieve an effective plasma level at a lower dose [14].

Myelosuppression is an independent adverse factor for achieving cytogenetic response with imatinib in patients with CML. These patients also have higher risk of relapses [15]. Imatinib therapy induced myelosuppression is noted to have worse prognosis and has poor response to therapy [16]. Conservative management with holding or decreasing imatinib lead to slow recovery of blood counts. Supportive care with blood transfusions and colony stimulating agents can help maintain the blood counts till marrow recovers.

## Conclusions

Pancytopenia is a rare but known complication of TKIs and should be kept in differential workup. Bone marrow biopsy is usually needed to evaluate for bone marrow aplasia and to rule out myelodysplastic syndrome, myelofibrosis, or transformation to acute leukemia. Treatment involves stopping the offending agent and giving time for bone marrow recovery. Supportive treatment with blood transfusion is indicated. Various degrees of cytopenia can present with tyrosine kinase therapy and regular follow up to monitor blood counts is necessary to modify or stop the offending agent on time. Given the scarcity of data to evaluate the degree of myelosuppression caused by Imatinib our case report highlights the need for increased research on myelosuppression with TKI therapy.
